# Supplementary material for: Comparison of Three Algorithms for Predicting Infarct Volume in Patients with Acute Ischemic Stroke by CT Perfusion Software: Bayesian, CSVD, and OSVD
Source: Diagnostics (Basel). 2023 May 20;13(10):1810. doi: 10.3390/diagnostics13101810 (PMC10217375; doi:10.3390/diagnostics13101810)
Supplement: Supplementary file 1 [file diagnostics-13-01810-s001.zip › diagnostics-2292832-supplementary.pdf]

Figure S1. The correlation between PIV and FIV of three algorithms in subgroup analysis.

A-C for Subgroup 1.a. Patients with conservative treatment and abundant collaterals (predicted infarct core volume & FIV)

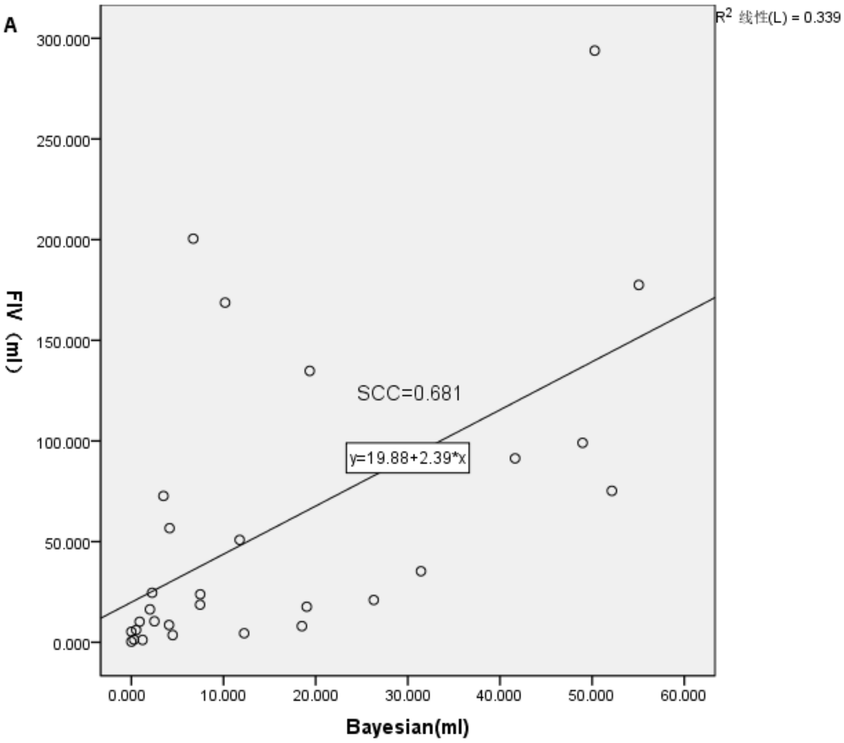

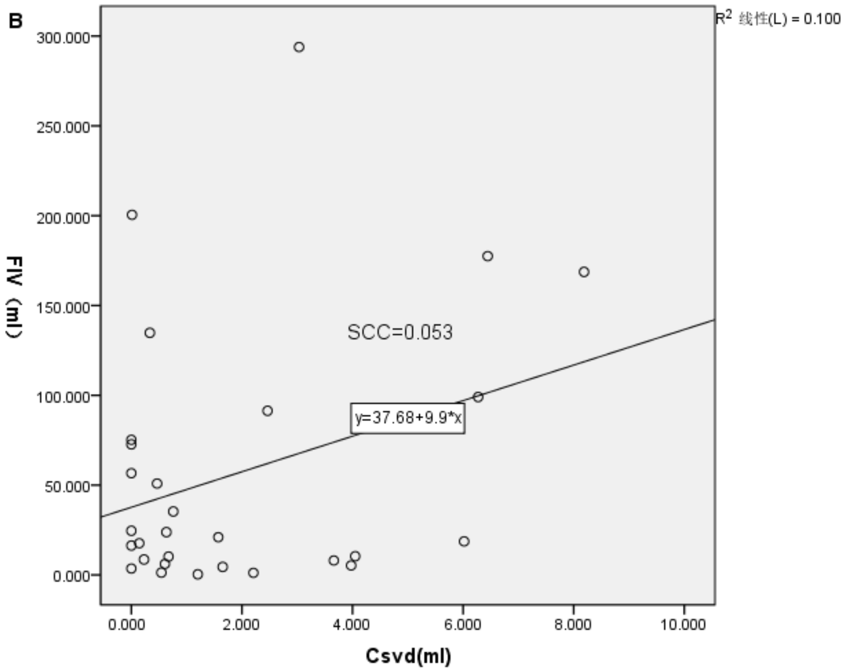

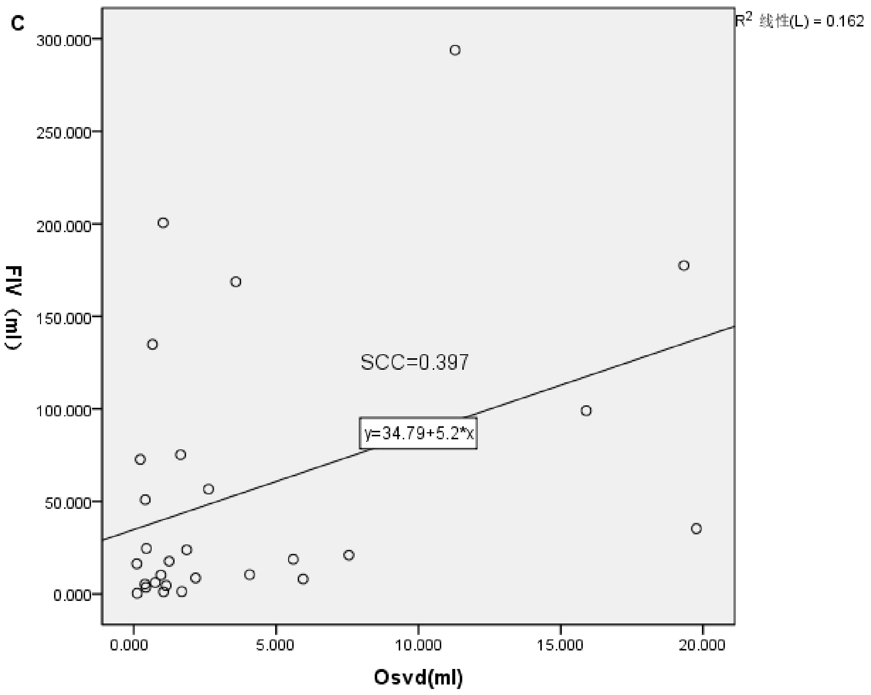
C
R^2 线性(L) = 0.162
300.000
250.000
200.000
150.000
100.000
50.000
0.000
FIV（ml）
SCC=0.397
y=34.79+5.2*x
0.000
5.000
10.000
15.000
20.000
Osvd(ml)

D-F for Subgroup 1.b. Patients with conservative treatment and abundant collaterals (predicted hypoperfusion area volume& FIV)

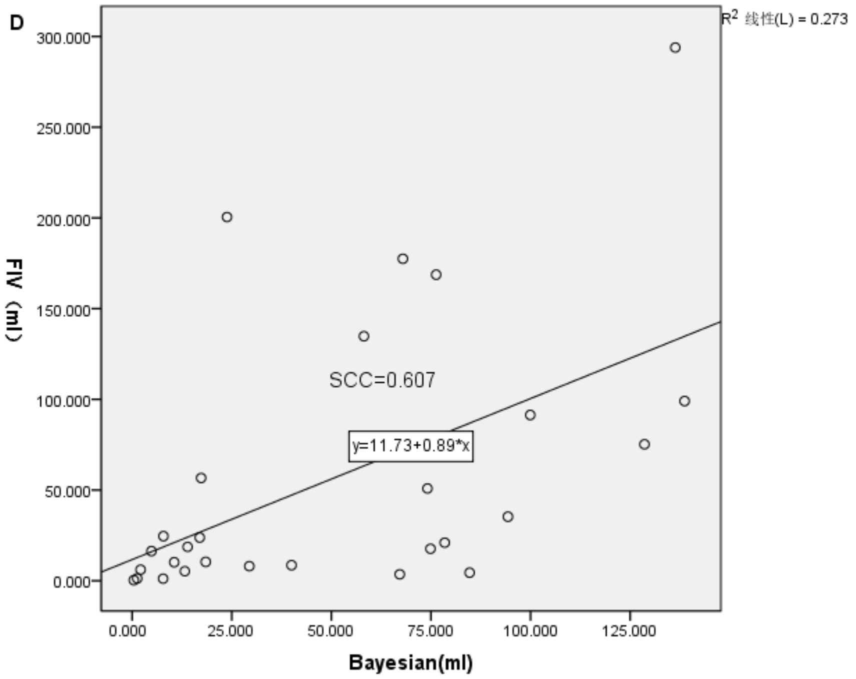

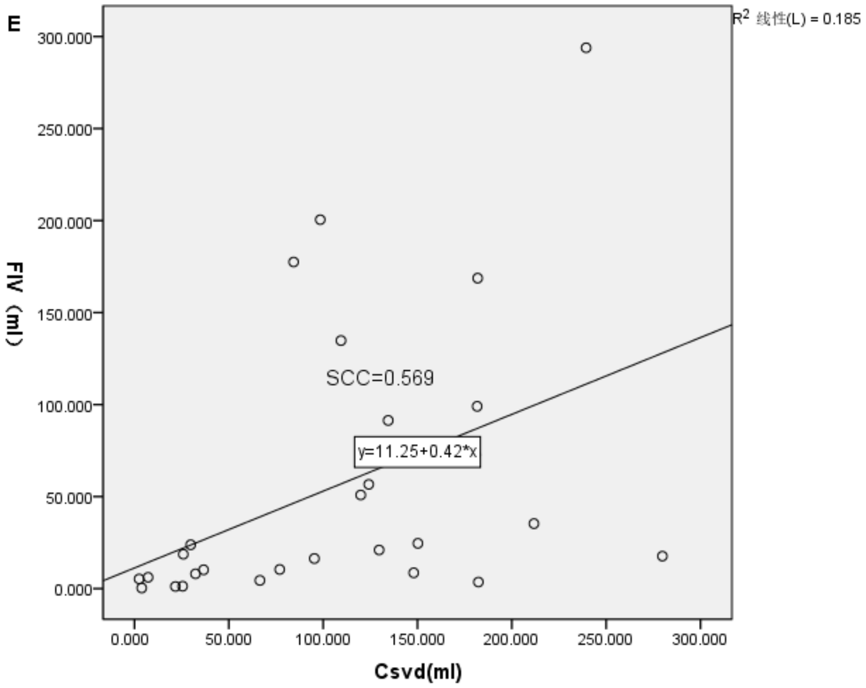

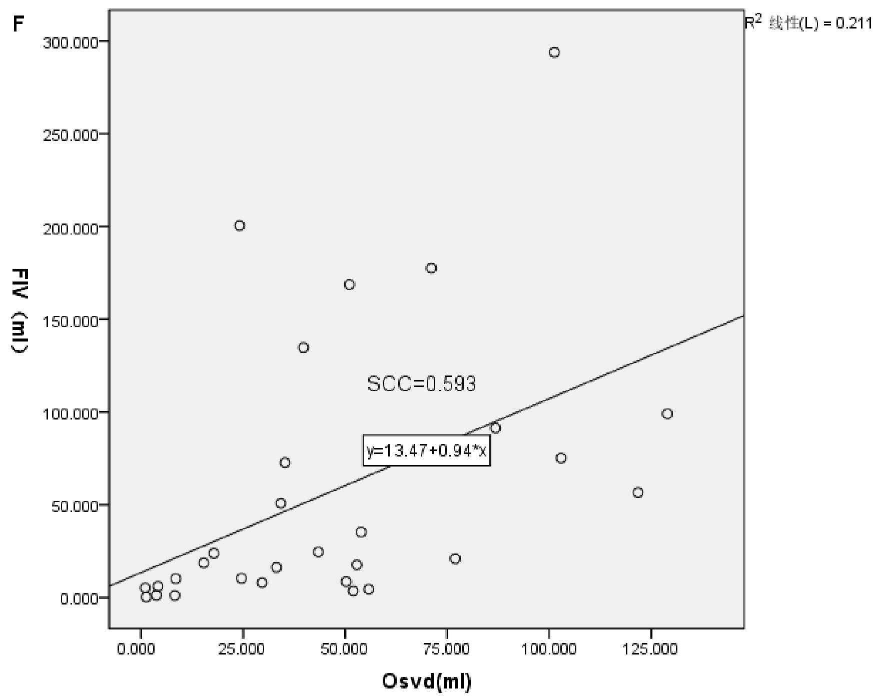

G-I for Subgroup 2. Patients with conservative treatment and poor collateral condition (predicted hypoperfusion volume& FIV)

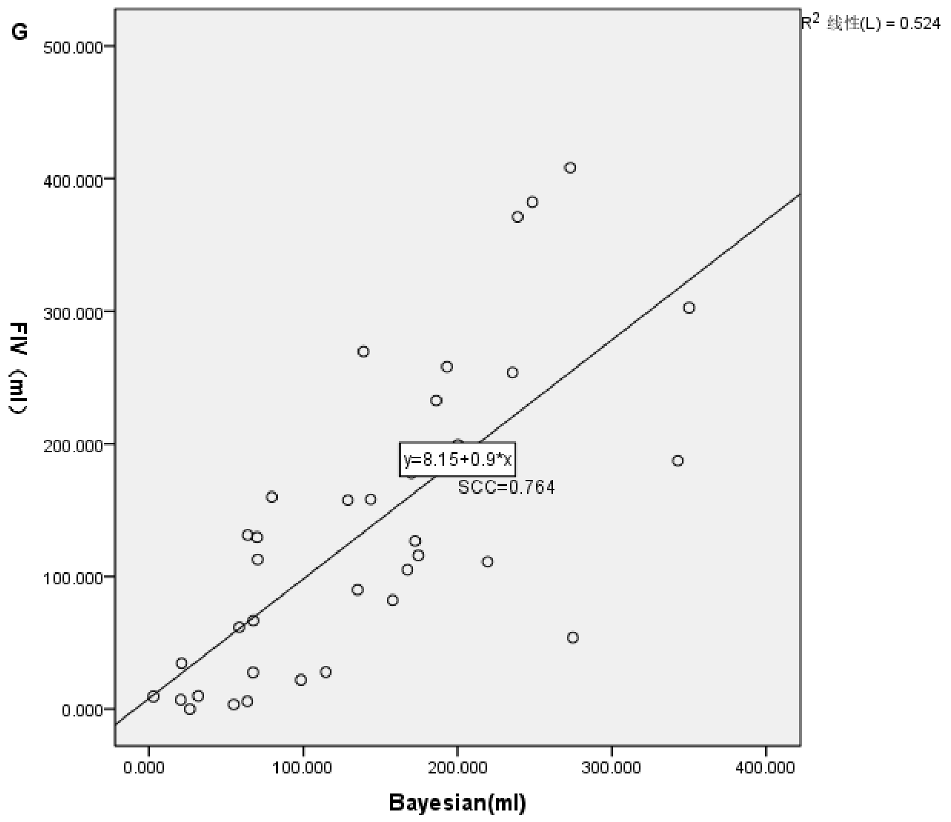

H

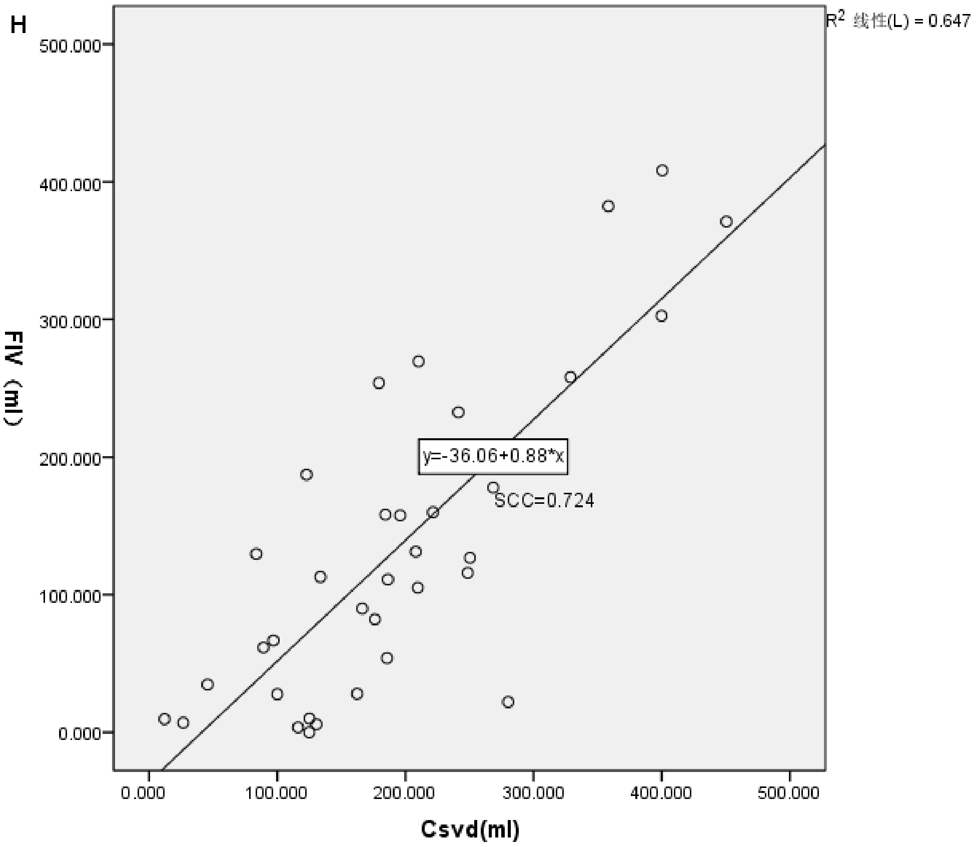
R2 线性(L) = 0.647
500.000
400.000
300.000
200.000
100.000
0.000
FIV（ml）
y=-36.06+0.88*x
SCC=0.724
0.000
100.000
200.000
300.000
400.000
500.000
Csvd(ml)

I

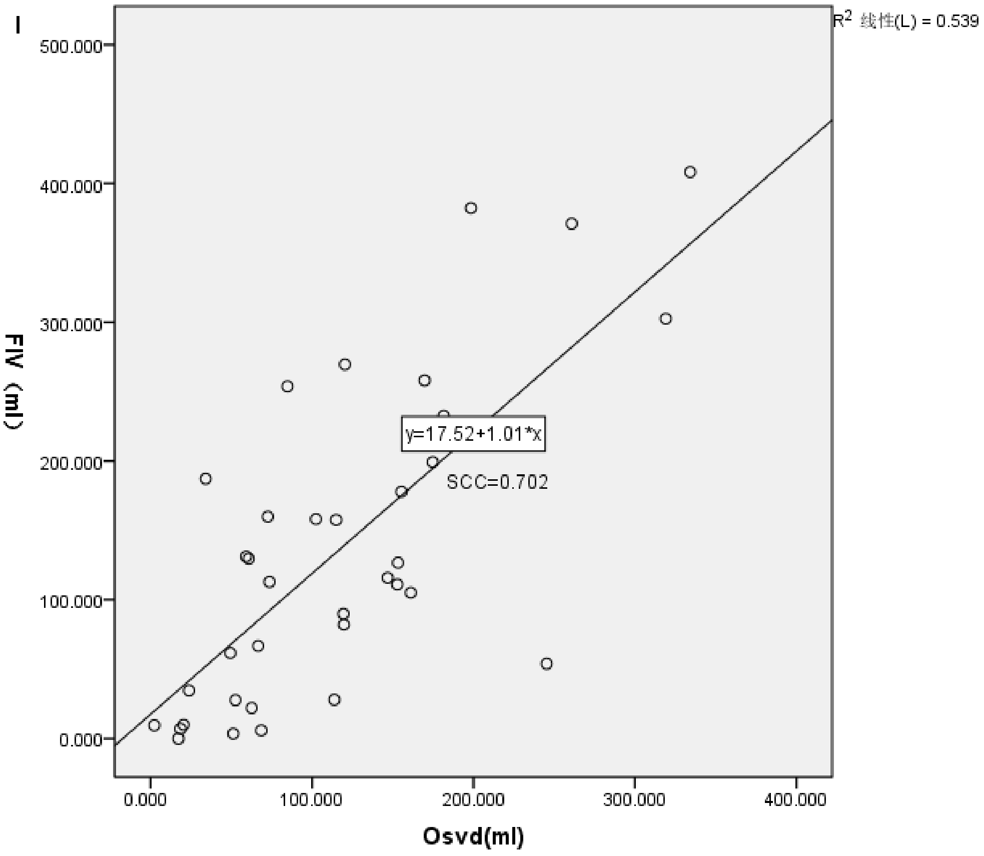
R2 线性(L) = 0.539
500.000
400.000
300.000
200.000
100.000
0.000
FIV（ml）
y=17.52+1.01*x
SCC=0.702
0.000
100.000
200.000
300.000
400.000
Osvd(ml)

A-C For subgroup 1 (with conservative treatment and abundant collaterals) to show the correlation between FIV and the predicted infarct core volume which were predicted by Bayesian, CSVD, and OSVD algorithms. D-F For subgroup 2 (with conservative treatment and abundant collaterals) to show the correlation between FIV and the predicted hypoperfusion area volume which were predicted by Bayesian, CSVD, and OSVD algorithms. G-I For subgroup 3 (with conservative treatment and poor collateral condition) to show the correlation between FIV and the predicted hypoperfusion area volume which were predicted by Bayesian, CSVD, and OSVD algorithms.
FIV, final infarct volume; SCC, spearman correlation coefficient; CSVD, cyclic singular value decomposition algorithms; OSVD, oscillatory exponential singular value decomposition algorithm.
